# Doxorubicin Impairs the Insulin-Like Growth Factor-1 System and Causes Insulin-Like Growth Factor-1 Resistance in Cardiomyocytes

**DOI:** 10.1371/journal.pone.0124643

**Published:** 2015-05-08

**Authors:** Patrizia Fabbi, Paolo Spallarossa, Silvano Garibaldi, Chiara Barisione, Marzia Mura, Paola Altieri, Barbara Rebesco, Maria Gaia Monti, Marco Canepa, Giorgio Ghigliotti, Claudio Brunelli, Pietro Ameri

**Affiliations:** 1 Research Center of Cardiovascular Biology, Department of Internal Medicine, University of Genova, Genova, Italy; 2 Antiblastic Drug Unit, IRCCS AOU San Martino-IST, Genova, Italy; 3 Department of Medical Translational Sciences, University of Napoli Federico II, Napoli, Italy; National Institutes of Health, UNITED STATES

## Abstract

**Background:**

Insulin-like growth factor-1 (IGF-1) promotes the survival of cardiomyocytes by activating type 1 IGF receptor (IGF-1R). Within the myocardium, IGF-1 action is modulated by IGF binding protein-3 (IGFBP-3), which sequesters IGF-1 away from IGF-1R. Since cardiomyocyte apoptosis is implicated in anthracycline cardiotoxicity, we investigated the effects of the anthracycline, doxorubicin, on the IGF-1 system in H9c2 cardiomyocytes.

**Methods and Results:**

Besides inducing apoptosis, concentrations of doxorubicin comparable to those observed in patients after bolus infusion (0.1-1 µM) caused a progressive decrease in IGF-1R and increase in IGFBP-3 expression. Exogenous IGF-1 was capable to rescue cardiomyocytes from apoptosis triggered by 0.1 and 0.5 µM, but not 1 µM doxorubicin. The loss of response to IGF-1 was paralleled by a significant reduction in IGF-1 availability and signaling, as assessed by free hormone levels in conditioned media and Akt phosphorylation in cell lysates, respectively. Doxorubicin also dose-dependently induced p53, which is known to repress the transcription of *IGF1R* and induce that of *IGFBP3*. Pre-treatment with the p53 inhibitor, pifithrin-α, prevented apoptosis and the changes in IGF-1R and IGFBP-3 elicited by doxorubicin. The decrease in IGF-1R and increase in IGFBP-3, as well as apoptosis, were also antagonized by pre-treatment with the antioxidant agents, N-acetylcysteine, dexrazoxane, and carvedilol.

**Conclusions:**

Doxorubicin down-regulates IGF-1R and up-regulates IGFBP-3 via p53 and oxidative stress in H9c2 cells. This leads to resistance to IGF-1 that may contribute to doxorubicin-initiated apoptosis. Further studies are needed to confirm these findings in human cardiomyocytes and explore the possibility of manipulating the IGF-1 axis to protect against anthracycline cardiotoxicity.

## Introduction

Cardiac toxicity occurs in a significant percentage of patients treated with drugs belonging to the anthracycline class, among which doxorubicin [1]. One of the features of anthracycline cardiotoxicity is the induction of apoptosis of both terminally differentiated cardiomyocytes and cardiac progenitor cells (CPCs), resulting in the loss of myocardial tissue and intrinsic regenerative capacity, respectively [2,3].

It has long been known that insulin-like growth factor-1 (IGF-1) protects against cardiomyocyte apoptosis [4]. More recently, IGF-1 has been specifically shown to enhance the proliferation and survival of CPCs [5,6]. IGF-1 may reach the heart via the circulation or may be secreted within the myocardium, thereby acting in an endocrine or paracrine/autocrine way, respectively. Regardless of the source, IGF-1 binds to type 1 IGF receptor (IGF-1R) to activate anti-apoptotic signaling pathways [7]. At the tissue level, six different IGF binding proteins (IGFBP) can modulate the action of IGF-1 by sequestering it away from IGF-1R [8]. In particular, IGFBP-3 is the most abundant IGFBP in the heart [9] and its transcriptional up-regulation may be sufficient to inhibit CPC-mediated cardiac regeneration [10].

There is evidence that doxorubicin impairs the response of cardiomyocytes to different growth factors and hormones, such as hepatocyte growth factor [11] and testosterone [12]. It has also been reported that IGF-1 fails to counteract apoptosis when cardiac myocytes are exposed to doxorubicin [13]. However, another group of investigators has found IGF-1 to inhibit apoptosis triggered by doxorubicin [14,15].

Here, we characterized the endogenous expression of IGF-1, IGF-1R, and IGFBP-3, as well as the effect of exogenous IGF-1, in H9c2 cells treated with doxorubicin, in order to better understand whether, to which extent, and how this chemotherapeutic agent alters IGF-1 anti-apoptotic activity on cardiomyocytes.

## Methods

### Cell culture and treatments

The embryonic rat cell line H9c2 was purchased from the American Type Culture Collection (ATCC, Rockville, MD, USA; CRL-1446) and cultured in Dulbecco’s modified Eagle’s medium supplemented with 10% fetal bovine serum, 4 mM glutamine, 100 IU/mL penicillin, and 100 μg/mL streptomycin at 37°C in a 5% CO_2_ humidified incubator. These cells exhibit features of both adult and immature embryonic cardiocytes [16]; in particular, they retain proliferative and differentiative potential similar to CPCs.

Cells were treated at a density of 60–70%. Apoptosis and expression of the IGF-1 axis components were evaluated 24 hours after adding doxorubicin and/or IGF-1. Doxorubicin (Adriblastina, Pfizer, New York, NY, USA) was provided by the Antiblastic Unit of the IRCCS AOU San Martino-IST University Hospital and used at 0.1, 0.5, or 1 μM, which correspond to the drug levels observed in patients after bolus infusion [1]. IGF-1 (PeproTech, Rocky Hill, NJ, USA) was also tested at three different concentrations: 100 ng/mL (10^–8^ M), as previously done in studies of doxorubicin-induced apoptosis [14,15], and 10 ng/mL (10^–9^ M) and 0.01 ng/mL (10^–12^ M), which approximate the amount of IGF-1 measured in heart lysates [17] and cardiac cell cultures [18], respectively. In some experiments, H9c2 cardiomyocytes were pre-treated with 5μM pifithrin-alpha (PFT-α; BIOMOL International, LP Plymouth Meeting, PA, USA) for 1 hour, 50 μM N-acetylcysteine (Sigma-Aldrich, St. Louis, MO, USA) for 1 hour, 20 μM dexrazoxane (Sigma-Aldrich) for 3 hours, or 10 μM carvedilol (a gift from Roche Diagnostics, Monza, Italy) for 1 hour.

To determine the effect on IGF-1 free levels and signaling, cells were incubated with 100 ng/mL IGF-1 for 2 hours or 5 minutes, respectively, after being treated with 0.1, 0.5, or 1 μM doxorubicin for 7 hours.

### Assessment of apoptosis

Apoptosis was investigated in three different ways: flow cytometry for annexin V/propidium iodide, terminal deoxynucleotidyl transferase (TdT)–mediated deoxyuridine triphosphate in situ DNA nick end labeling (TUNEL), and assessment of caspase 3/7 activity.

For annexin V/propidium iodide staining, 1x10^6^cells were washed twice with PBS and resuspended in 100 μL of binding buffer of the Alexa Fluor 488 Annexin V/Dead Cell Apoptosis Kit (Invitrogen-Life Technologies Ltd, Paisley, UK). Five μL of annexin V and 1 μL of propidium iodide were added to the cell suspension and incubated for 10 minutes in the dark at room temperature. After adding another 250 μL of binding buffer to every sample, annexin V fluorescence was measured on an Attune Acoustic Focusing Cytometer (Life Technologies Ltd).

The TUNEL assay (Promega, Madison, WI, USA) was performed according to the manufacturer’s instructions on cells that had been grown and treated on Histobond slides, fixed in methanol, and permeabilized with 0.2% Triton X-100. The percentage of nuclei with fragmented DNA was counted by direct observation of a total of 500 cells for each condition with a NIKON eclipse 80i microscope at ×60 magnification. Negative controls without the TdT enzyme were included.

Caspase-3 and caspase-7 activity was analyzed by means of the CellEvent Caspase-3/7 Green Flow Cytometry Assay Kit (Life Technologies Ltd), which is based on cleavage of a synthetic substrate by activated caspase-3 and caspase-7, with ensuing release of a DNA-binding dye that labels apoptotic cells. The fluorogenic signal of the dye was quantified by using the Attune Acoustic Focusing Cytometer.

### Real-time RT-PCR

Total RNA was extracted by using the Quick-RNA MiniPrep kit (Zymo Research, Irvine, CA, USA) and reverse transcribed to cDNA by means of Euroscript Reverse Transcriptase (Euroclone Group, Milano, Italy). RT-PCR was performed with primers and probes purchased from IDT—Integrated DNA Technologies (Coralville, IA, USA; assay ID Rn.PT.58.38098384, Rn.PT.58.18302903, Rn.PT.58.36919417, and Rn.PT.58.35727291 for *Igf1r*, *Igfbp3*, *Igf1*, and *Gapdh*, respectively). Samples were amplified by using a TaqMan universal mastermix (Life Technologies Ltd) on a CFX96 Touch RT-PCR Detection Systems (Bio-Rad, Hercules, CA, USA). The expression of the genes of interest was normalized against that of *Gapdh* and analyzed with the comparative Ct method for quantification of transcripts.

### Western blotting

H9c2 cells were lysed on ice in lysis buffer (50 mM Tris HCl pH 8.0, 150 mM NaCl, 5 mM EDTA, 1% NP40) with 1 mM phenylmethylsulfonyl fluoride and a cocktail of protease and phosphatase inhibitors (Sigma Aldrich). Proteins were run on a 8–16% tris-glycine gel (Thermo Fisher Scientific, Waltham, MA, USA) and then transferred onto polyvinylidene difluoride membranes (Bio-Rad). After blocking with 5% non-fat dry milk in TBS-tween for 1 hour at room temperature, membranes were incubated overnight with the following rabbit primary antibodies: polyclonal anti-IGF-1R, anti-IGFBP-3, anti-GAPDH, and anti-p53 (all from Santa Cruz Biotechnology, Dallas, TX, USA); polyclonal anti-total Akt and monoclonal anti-phosphorylated Akt (Ser473), anti-phosphorylated insulin receptor substrate-1 (IRS-1) (Ser612), and anti-total IRS-1 (all from Cell Signaling Technologies, Danvers, MA, USA). To detect the bound primary antibody, a peroxidase-coupled anti-rabbit secondary antibody was used (Amersham Life Sciences, Arlington Heights, IL, USA). The intensity of the protein bands was quantified by densitometry with a chemiluminescence lighting system (Luminata Classico, Millipore, Billerica, MA, USA).

### Quantification of free IGF-1

In any moment, very few IGF-1 is not combined with IGFBP or bound to IGF-1R [19]. To accurately quantify such free IGF-1, conditioned media were first filtered through Centriprep Centrifugal Filter Units (Millipore), which separate low molecular weight molecules, among which IGF-1, from bigger ones, including IGF-1/IGFBP complexes, and concentrate them. Free IGF-1 was then measured in the low molecular weight fraction by radioactive immunoassay (DIAsource ImmunoAssays, Nivelles, Belgium). The results were divided by the concentration factor to interpolate the actual values.

### Analysis of oxidative stress

To estimate the intracellular production of reactive oxygen species, 20 μM 2´,7´-dichlorodihydrofluorescein (DCFH) was added to H9c2 cardiomyocytes after one hour-exposure to doxorubicin and incubated for 30 minutes at 37°C. The amount of fluorescent 2´,7´-dichlorofluorescein formed by oxidation of DCFH was determined by cytometry with the Attune Acoustic Focusing apparatus.

### Statistical analysis

Data are presented as mean and SEM of at least three independent replicates for each experiment. Comparisons were drawn by unpaired t-test or ANOVA. Statistical significance was set at P <0.05.

## Results

### Doxorubicin-initiated apoptosis is associated with IGF-1R/IGFBP-3 modulation and IGF-1 resistance

**Fig 1:**
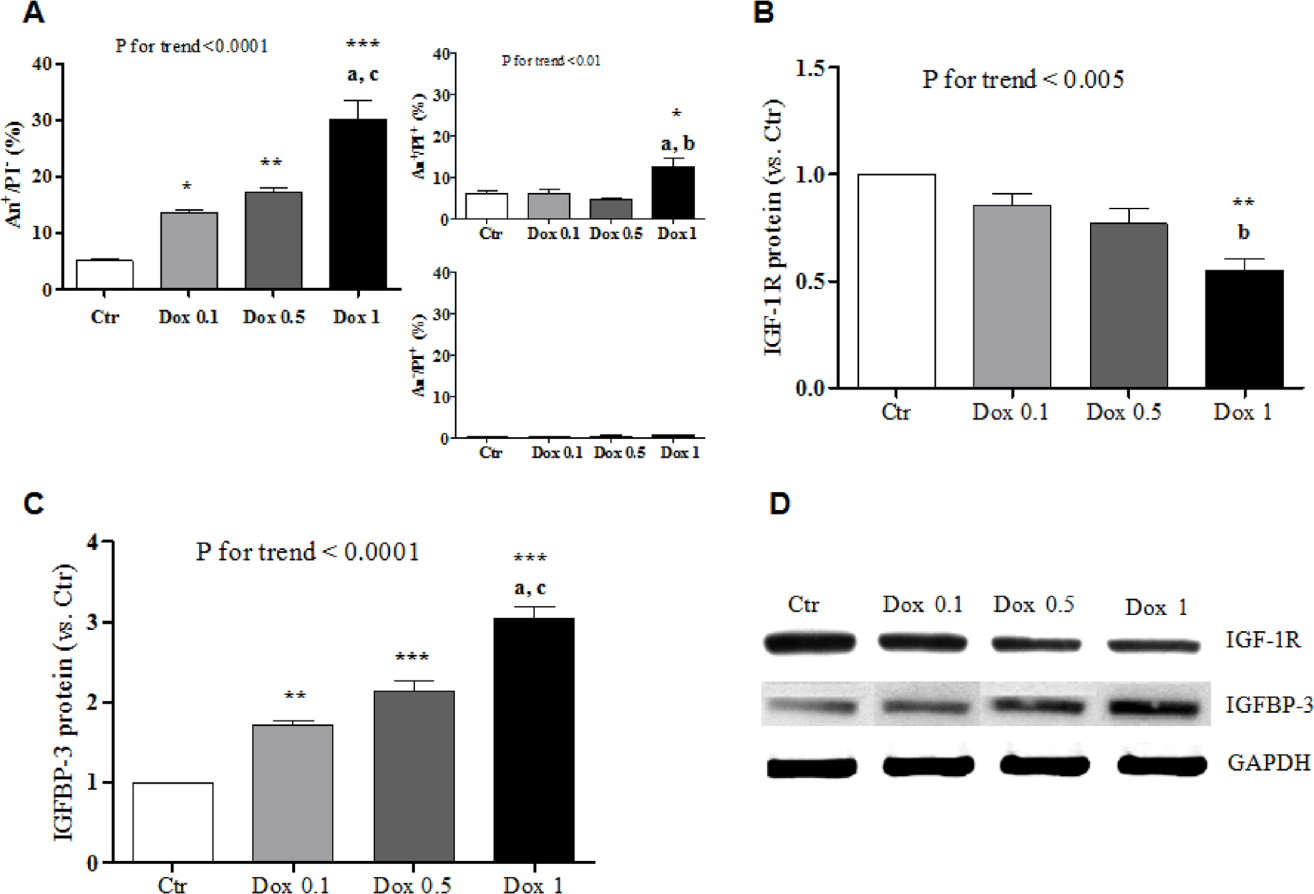
Doxorubicin stimulates apoptosis and modulates IGF-1R/IGFBP-3 expression in H9c2 cells. Frequency of apoptotic cells (A) and IGF-1R (B) and IGFBP-3 (C) expression (densitometry of western blot bands) 24 hours after no treatment (Ctr) or incubation of H9c2 cardiomyocytes with 0.1, 0.5, or 1 μM doxorubicin (Dox). A representative western blot for IGF-1R and IGFBP-3 is shown in (D). *, P <0.05 vs. Ctr; **, P <0.01 vs. Ctr; ***, P <0.001 vs. Ctr. a, P <0.01 vs. Dox 0.5; b, P <0.05 vs. Dox 0.1; c, P <0.001 vs. Dox 0.1.

Doxorubicin concentration-dependently caused apoptosis of H9c2 cells (Fig 1A). Exposure to the drug also resulted in a decrease in *Igf1r* mRNA and in an increase in the *Igfbp3* one (S1 Fig). The amount of IGF-1R and IGFBP-3 protein was similarly modified by doxorubicin in a dose-dependent manner (Fig 1B–1D). Conversely, *Igf1* was not expressed in H9c2 cardiomyocytes, nor was it affected by treatment with doxorubicin.

**Fig 2:**
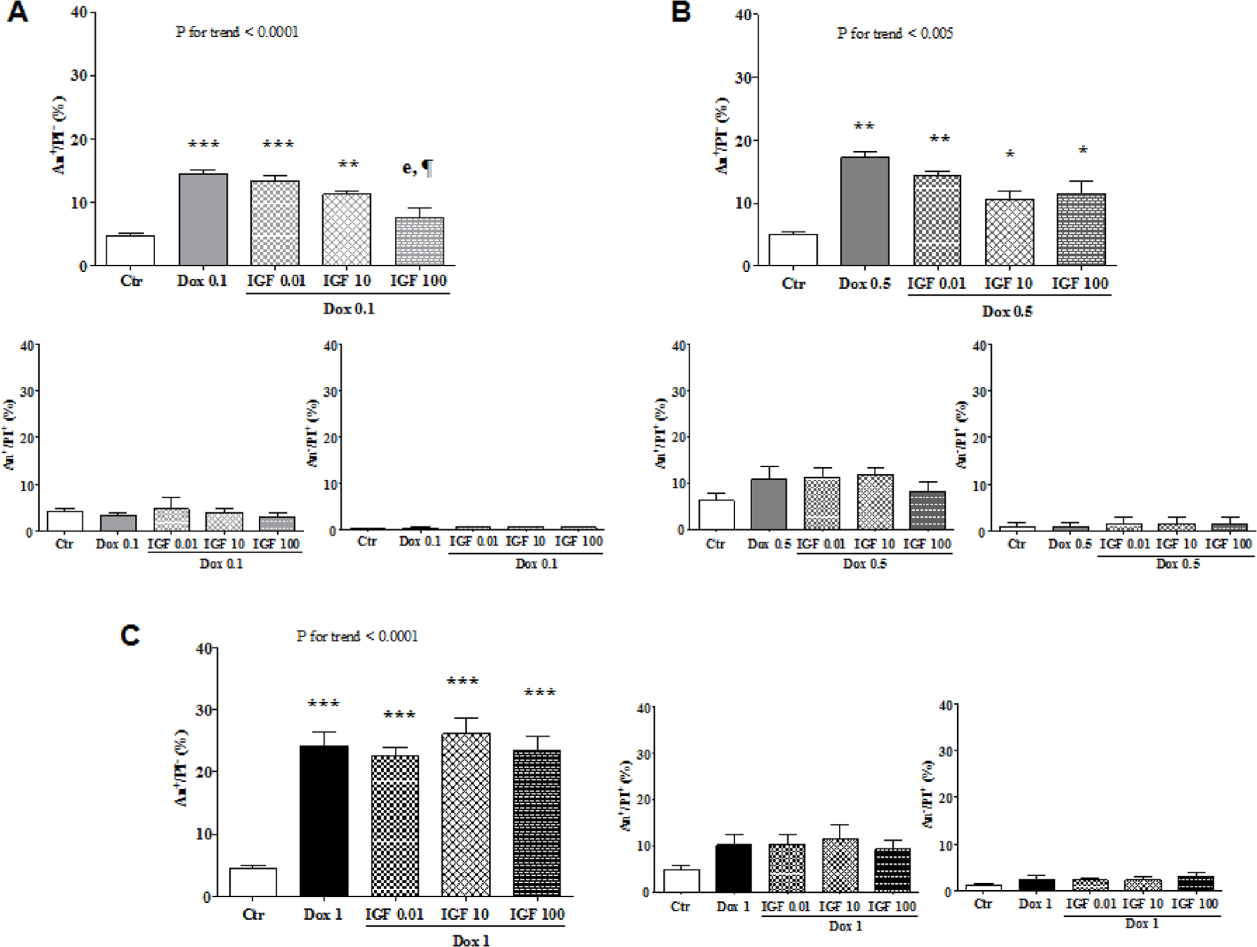
Effect of exogenous IGF-1 on doxorubicin-induced apoptosis of H9c2 cells: annexin V/propidium iodide. Frequency of apoptotic cells, as assessed by annexin V/propidium iodide staining, 24 hours after no treatment (Ctr) or incubation of H9c2 cardiomyocytes with doxorubicin (Dox) ± IGF-1 at the indicated concentrations. *, P <0.05 vs. Ctr; **, P <0.01 vs. Ctr; ***, P <0.001 vs. Ctr. e, P <0.01 vs. Dox 0.1; ¶, P <0.05 vs. IGF-1 0.01.

**Fig 3:**
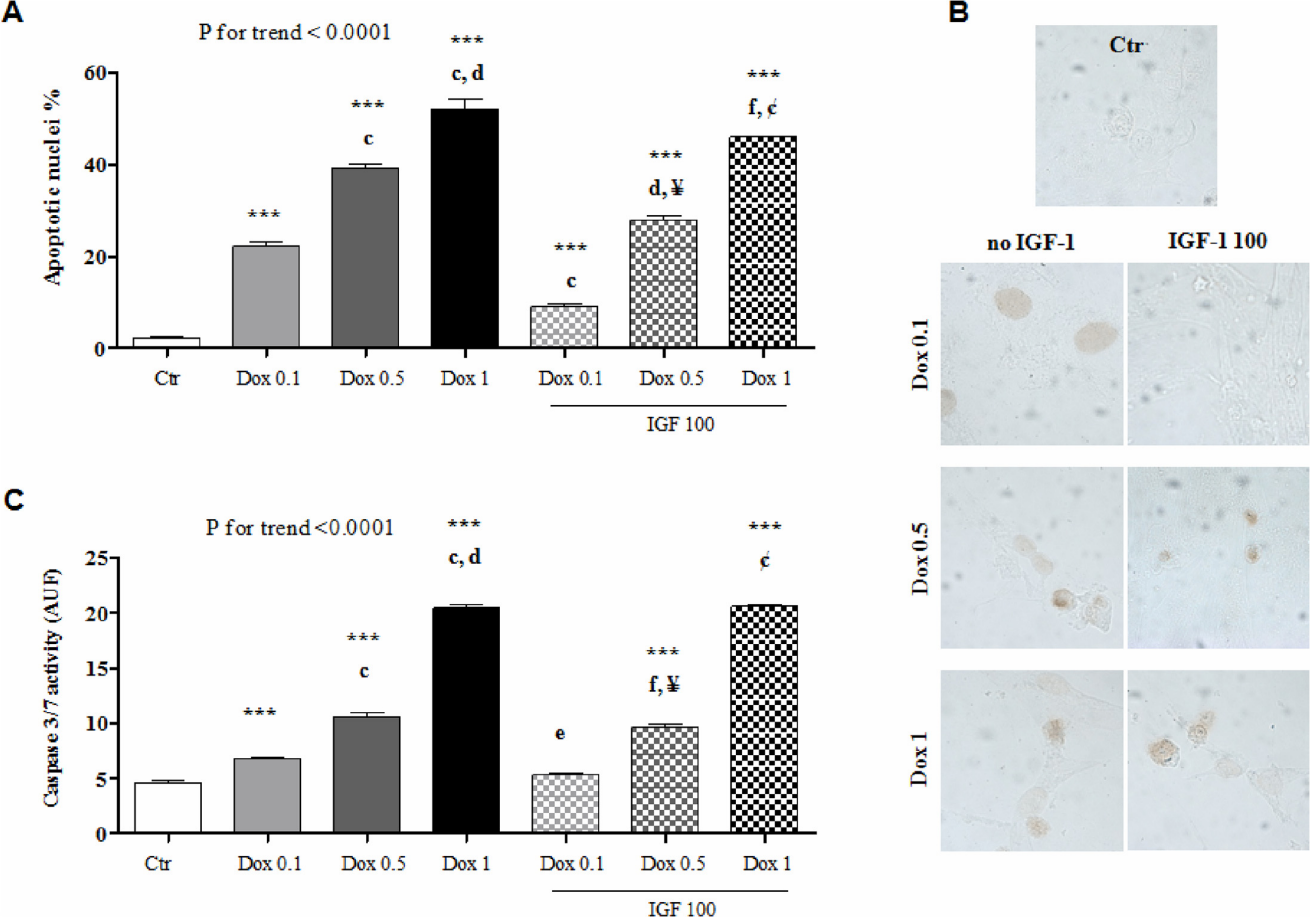
Effect of exogenous IGF-1 on doxorubicin-induced apoptosis of H9c2 cells: TUNEL and caspase 3/7 activity. Frequency of apoptotic cells, as assessed by TUNEL (A; representative microphotographs are shown in B) and fluorescence (AUF) produced by the cleavage of a substrate of activated caspase 3/7 (C), 24 hours after no treatment (Ctr) or incubation of H9c2 cardiomyocytes with doxorubicin (Dox) ± IGF-1 at the indicated concentrations. ***, P <0.001 vs. Ctr. c, P <0.001 vs. Dox 0.1; d, P <0.001 vs. Dox 0.5; e, P <0.01 vs. Dox 0.1; f, P <0.05 vs. Dox 0.5. ¥, P <0.001 vs. Dox 0.1 + IGF-1 100; ¢, P <0.001 vs. Dox 0.1 + IGF-1 100 and Dox 0.5 + IGF-1 100.

Exogenous IGF-1 was capable to rescue H9c2 cardiomyocytes from apoptosis stimulated by 0.1 and 0.5 μM doxorubicin, as assessed by annexin V/propidium iodide staining (Fig 2A and 2B). By contrast, IGF-1 was no longer effective in the presence of 1 μM doxorubicin (Fig 2C). These results were substantiated by evaluating TUNEL positivity (Fig 3A and 3B) or caspase 3/7 activity (Fig 3C) in cells incubated with the highest tested concentration of IGF-1 (100 ng/mL) along with 0.1, 0.5, or 1 μM doxorubicin.

**Fig 4:**
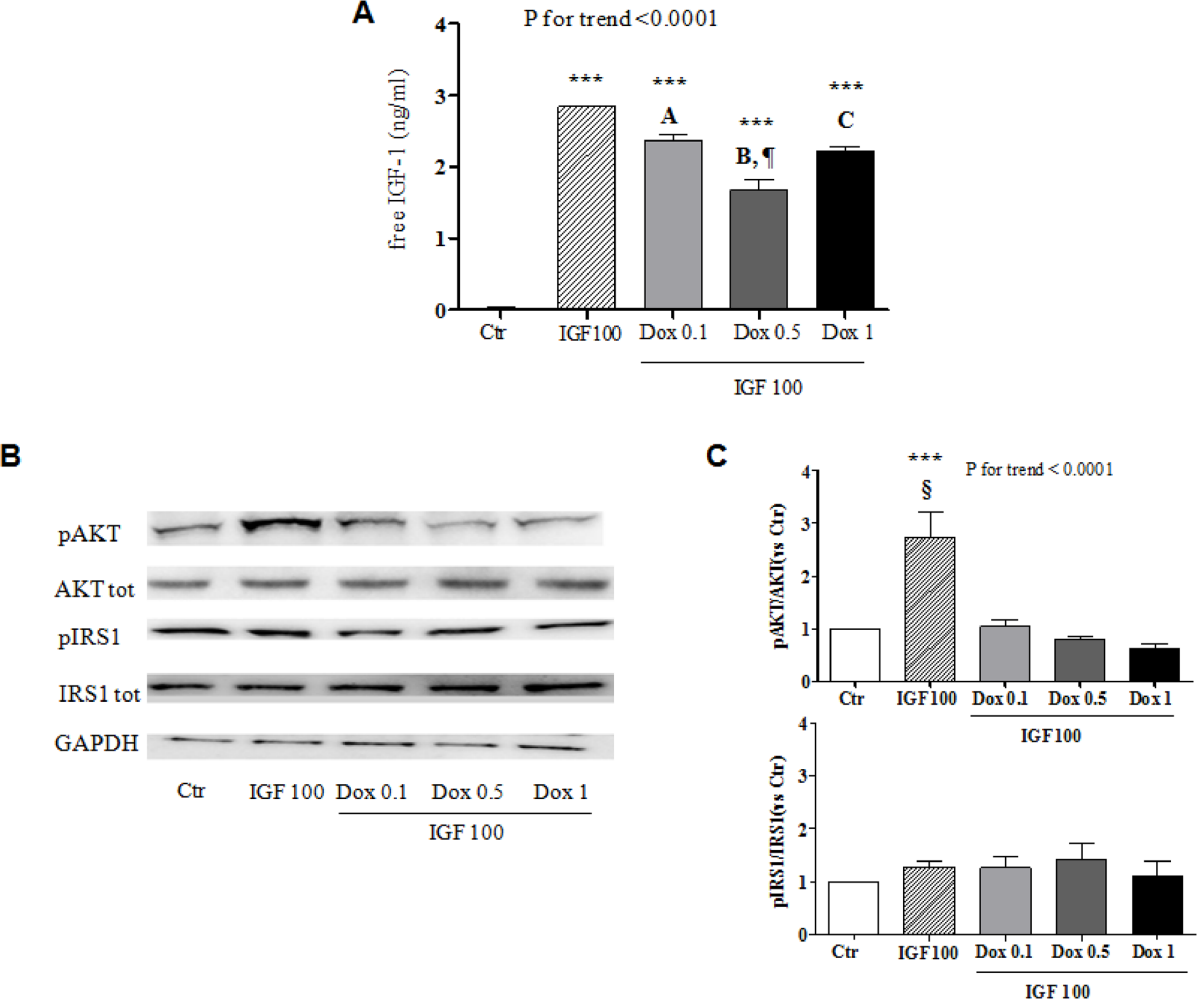
Doxorubicin affects IGF-1 free levels and intracellular signaling. (A) Concentrations of free IGF-1 measured in culture media two hours after adding 100 ng/ml IGF-1 to H9c2 cardiomyocytes that had not been treated (Ctr) or had been incubated with 0.1, 0.5, or 1 μM doxorubicin (Dox). (B and C) IGF-1 stimulated phosphorylation of Akt and IRS-1 (B, representative western blots; C, band densitometry) in H9c2 cardiomyocytes that had not been treated (Ctr) or had been incubated with 0.1, 0.5, or 1 μM Dox. ***, P <0.001 vs. Ctr. A, P <0.05 vs. IGF-1 100; B, P <0.001 vs. IGF-1 100; C, P <0.01 vs. IGF-1 100. ¶, P <0.01 vs. Dox 0.1 + IGF-1 100 and Dox 1 + IGF-1 100; §, P <0.001 vs. Dox 0.1 + IGF-1 100, Dox 0.5 + IGF-1 100, Dox 1 + IGF-1 100.

As only IGF-1 that is not bound to IGFBP engages IGF-1R and exert biological effects [19], we determined the amount of free hormone two hours after adding 100 ng/mL IGF-1 to the culture media of doxorubicin-treated or control cardiomyocytes. As expected [18,19], the measured values were very small, being the result of IGF-1 degradation, sequestration by IGFBP-3 (and possibly other IGFBP), and interaction with IGF-1R that had occurred over the two hours. Consistent with the observed up-regulation of IGFBP-3 (Fig 1C), free IGF-1 levels progressively decreased in the media of cells that had been pre-incubated with 0.1 or 0.5 μM doxorubicin compared to untreated ones (Fig 4A). Following exposure to 1 μM doxorubicin, the concentration of free IGF-1 was also lower than in control media, but higher than the one reached with 0.5 μM doxorubicin. This U-shape trend may be explained by the fact that 1 μM doxorubicin not only induced IGFBP-3, but also significantly down-regulated IGF-1R (Fig 1B), thereby leading to the redistribution of free IGF-1 from the cell membrane to the culture medium.

Akt is a main mediator of IGF-1 pro-survival intracellular signaling [7]. In agreement with the diminished availability and impaired anti-apoptotic action of IGF-1, pre-exposure to doxorubicin profoundly inhibited the phosphorylation of Akt elicited by IGF-1 (Fig 4B and 4C). Serine phosphorylation of IRS-1, an adaptor protein that lies downstream of IGF-1R, may interrupt IGF-1 signaling cascade [7], but this was not the case with doxorubicin in H9c2 cells (Fig 4B and 4C).

### Doxorubicin modulates IGF-1R and IGFBP-3 via p53

**Fig 5:**
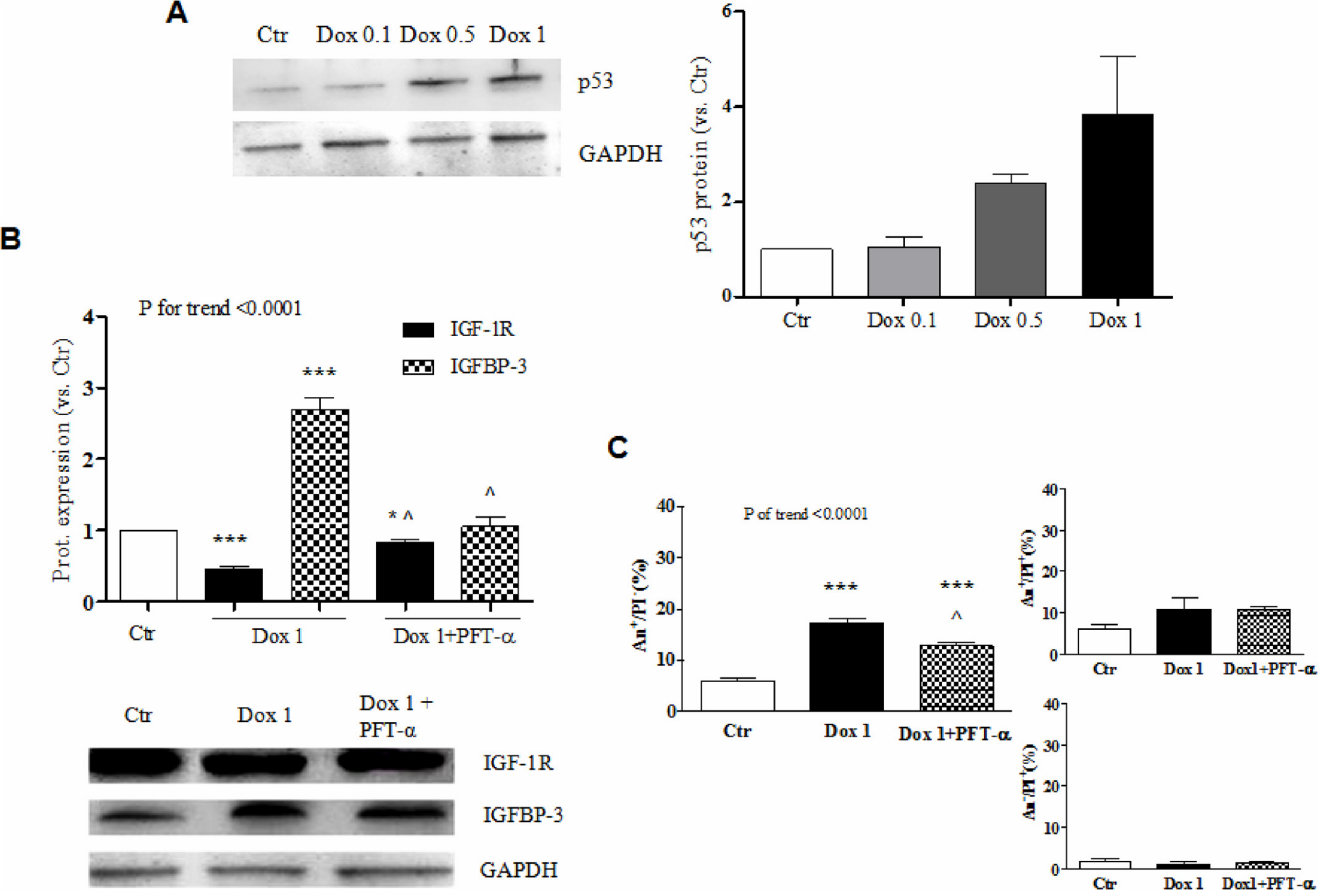
Involvement of p53 in the change in IGF-1R /IGFBP-3 levels caused by doxorubicin. (A) Representative western blot and band densitometry for p53 24 hours after no treatment (Ctr) or incubation of H9c2 cardiomyocytes with 0.1, 0.5, or 1 μM doxorubicin (Dox). (B and C) IGF-1R/IGFBP-3 expression (band densitometry and representative western blot, B) and annexin V/propidium iodide positivity (C) in H9c2 cardiomyocytes untreated or exposed to 1 μM Dox with or without pre-treatment with PFT-α. *, P <0.05 vs. Ctr; ***, P <0.001 vs. Ctr. ^, P <0.01 vs. Dox 1.

Since wild-type p53 is a transcriptional repressor of *IGF1R* [20] and an inducer of *IGFBP3* [21], we reasoned that the modifications in IGF-1R and IGFBP-3 levels triggered by doxorubicin might be mediated by p53. Indeed, doxorubicin dose-dependently caused the accumulation of p53 in H9c2 cells (Fig 5A), as already demonstrated [22]. To verify this hypothesis, cardiomyocytes were treated with 1 μM doxorubicin, which had caused the most intense perturbation of IGF-1R and IGFBP-3 expression, after being incubated with the p53 inhibitor, PFT-α. Pretreatment with PFT-α prevented the decrease in IGF-1R and the increase in IGFBP-3 induced by doxorubicin (Fig 5B). In addition, it reduced apoptosis (Fig 5C).

### Antioxidants antagonize doxorubicin-induced apoptosis and IGF-1R/IGFBP-3 modulation

**Fig 6:**
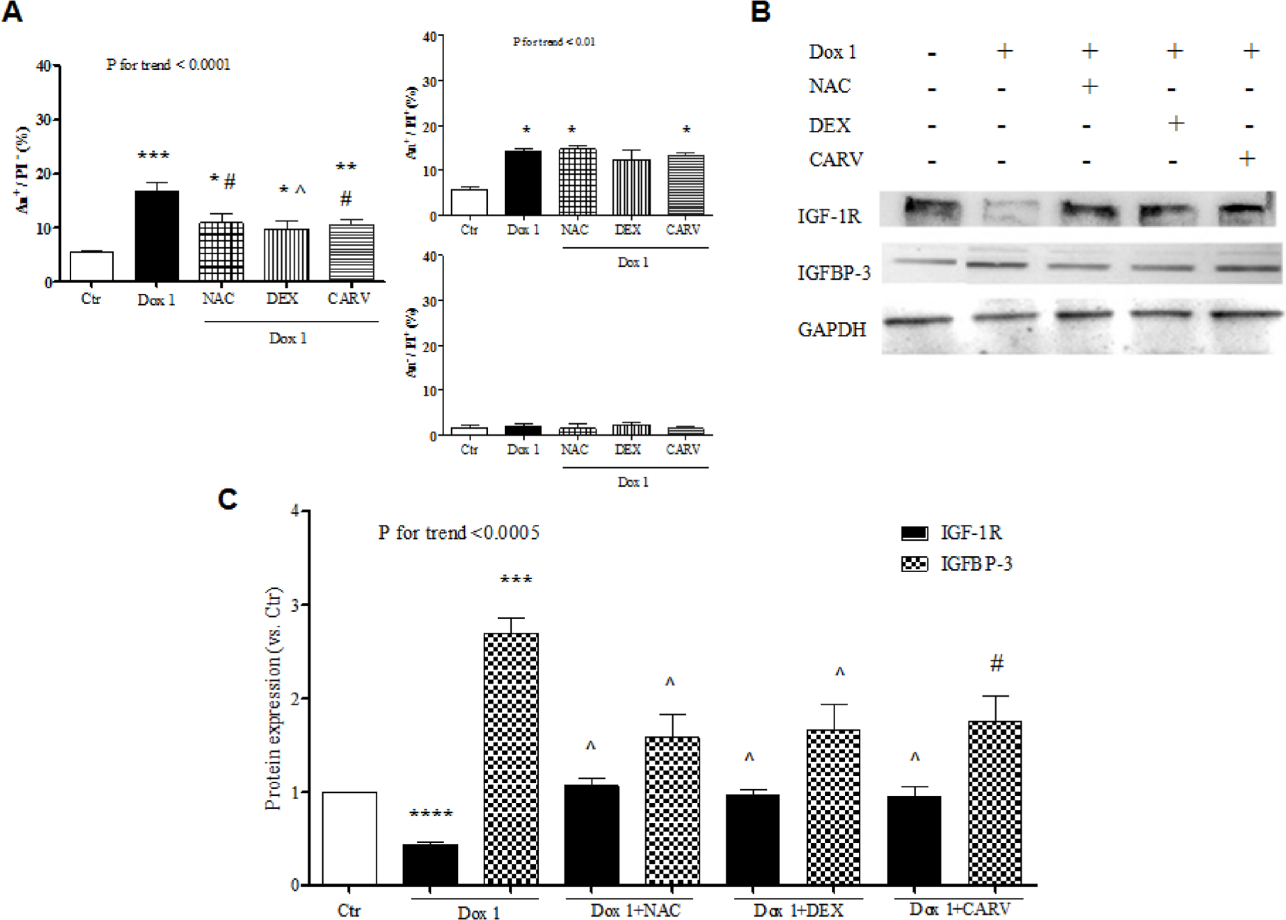
Antioxidants reverse doxorubicin-initiated apoptosis and IGF-1R /IGFBP-3 perturbation. Frequency of apoptotic cells (A) and IGF-1R and IGFBP-3 expression (representative western blot in B and densitometry of western blot bands in C) 24 hours after no treatment (Ctr) or incubation of H9c2 cardiomyocytes with 1 μM doxorubicin (Dox) with or without pre-treatment with N-acetylcysteine (NAC), dexrazoxane (DEX), or carvedilol (CARV). *, P <0.05 vs. Ctr; **, P <0.01 vs. Ctr; ***, P <0.001 vs. Ctr; ****, P <0.0001 vs. Ctr. #, P <0.05 vs. Dox 1; ^, P <0.01 vs. Dox 1.

Oxidative stress is known to be a major mechanism by which doxorubicin damages cardiomyocytes [23]. Consistently with our previous results [24], apoptosis triggered by 1 μM doxorubicin was associated with a significant increase in dichlorofluorescein production (S2 Fig). Pretreatment with the antioxidants, N-acetylcysteine, dexrazoxane, and carvedilol, diminished the oxidation of DCFH (S2 Fig) and protected cells against doxorubicin-elicited apoptosis (Fig 6A). Interestingly, N-acetylcysteine, dexrazoxane, and carvedilol also counteracted the changes in IGF-1R and IGFBP-3 secondary to doxorubicin exposure (Fig 6B and 6C).

## Discussion

A number of tumors, including common ones such as those of the breast or lymphomas, are treated with anthracyclines and especially doxorubicin [1]. Unfortunately, up to one in five patients develop some degree of cardiac toxicity following anthracycline chemotherapy, varying from subclinical left ventricular dysfunction to overt congestive heart failure [25]. Although the pathogenesis of this cardiotoxicity is multifactorial [23], experimental data indicate that apoptosis of cardiac cells may play an important role [26]. Remarkably, anthracycline-initiated apoptosis involves not only terminally differentiated cardiomyocytes, but also CPCs [2,3]. By depleting the CPC compartment, anthracyclines may impede the cardiac regeneration process and, thus, make the loss of cardiac tissue permanent [2].

The present study shows that, in H9c2 cells, doxorubicin causes a condition of resistance to IGF-1, an established anti-apoptotic factor for both cardiomyocytes and CPCs [4–7]. The responsiveness to IGF-1 appears to be compromised as a result of the down-regulation of IGF-1R, through which IGF-1 acts, and the up-regulation of IGFBP-3, which instead reduces the amount of IGF-1 available to interact with IGF-1R. Of note, IGFBP-3 can also cause apoptosis via IGF-1 independent mechanisms [27]. The variations in IGF-1R and IGFBP-3 are at least in part due to the accumulation and activation of p53, a key player in cardiac cell apoptosis prompted by anthracyclines. Indeed, upon exposure to doxorubicin the hearts of p53 knockout mice display significantly less apoptotic cells than their wild-type counterparts [28] and in our experiments p53 inhibition by PFT-α antagonized doxorubicin-induced apoptosis (Fig 5). Therefore, we hypothesize that the loss of sensitivity to IGF-1 and the increase in IGFBP-3 production are effectors of p53-dependent cardiomyocyte apoptosis elicited by anthracyclines.

Historically, it has been postulated that generation of reactive oxygen species (ROS) is the most upstream event in the cascade of intracellular alterations underlying anthracycline cardiotoxicity [1] and there have been reports that p53 induction is secondary to oxidative stress in cardiomyocytes treated with doxorubicin [22,29]. However, recent research work suggests that blockade of the activity of topoisomerase-IIβ is at the origin of anthracycline damage to cardiomyocytes [30] and is followed by p53 activation on the one side and ROS formation on the other one, the two phenomena enhancing each other [31]. Thus, our finding that pre-treatment with anti-oxidants abrogated doxorubicin effect on IGF-1R and IGFBP-3 expression may be ascribed to the induction of p53, but also to some oxidative stress-dependent, p53-independent pathway(s). This may be particularly true for the modulation of IGF-1R, which was completely prevented by anti-oxidants, but not by PFT-α (Figs 5 and 6).

Importantly, IGF-1 exerts other actions on cardiomyocytes and CPCs, such as stimulation of hypertrophy [32] and proliferation [5], respectively, that support the adaptation of the heart to injury. Although not assessed in this study, these cardioprotective effects are also likely to be hindered by doxorubicin.

Unlike *Igf1r* and *Igfbp3*, *Igf1* was not expressed by both unstimulated and doxorubicin-treated H9c2 cells. This is in agreement with previous investigations demonstrating that stromal cells, especially fibroblasts, mainly account for intramyocardial IGF-1 [32,33]. However, synthesis of IGF-1 by CPCs has been described in other experimental systems [6] and may contribute to the pool of the hormone within the cardiac tissue, even though to a minor extent. Moreover, a certain amount of IGF-1 is brought to the heart by the bloodstream [8].

This work also reconciles the results of earlier studies. Wang and colleagues reported that 100 ng/mL IGF-1 protected H9c2 cells from apoptosis induced by 0.5 μM doxorubicin [14,15], while Morales et al. did not observe any reduction in apoptosis when IGF-1 was added to 1 μM doxorubicin for 24 hours [13]. Moreover, silencing of IGFBP-3 by antisense oligonucleotides was found to inhibit doxorubicin-triggered apoptosis [34]. All these results are consistent with ours. Decreased expression of IGF-1R in response to doxorubicin has also been recently described in CPCs obtained from the atria of patients undergoing cardiac surgery [3], suggesting that the apoptosis experiments carried out here may yield similar results if performed with human cardiomyocytes.

The involvement of IGF-1R and IGFBP-3 dysregulation in anthracycline cardiotoxicity raises the concept that this latter may be diminished or even avoided by interventions that favorably influence the cardiac IGF-1 system. With this respect, it is interesting that angiotensin-converting enzyme inhibition, which has yielded promising results in clinical trials for primary and secondary prevention of anthracycline cardiotoxicity [31], has also been shown to up-regulate IGF-1R in the murine heart [35]. On the other hand, factors that reduce IGF-1R could accentuate cardiotoxicity of anthracyclines. For instance, high-glucose culture conditions lead to the down-regulation of cardiomyocyte IGF-1R via p53, similarly to doxorubicin [36], in agreement with the increased risk of anthracycline cardiotoxicity faced by oncological patients who also suffer from diabetes [25].

The limitations of the present work must be acknowledged. First, we did not examine whether doxorubicin also affects the expression of IGFBPs other than IGFBP-3. Second and most important, the experiments were done with the H9c2 cell line, which has features in between CPCs and differentiated cardiomyocytes, and did not include the evaluation of IGF-1 and IGFBP secretion by cardiac fibroblasts following exposure to doxorubicin. Future studies should be carried out with primary cardiomyocytes, CPCs, and fibroblasts in order to thoroughly characterize the effect of anthracyclines on the autocrine and paracrine IGF-1/IGF-1R/IGFBP axes involving cardiac cells.

## Conclusions

By altering the expression of IGF-1R and IGFBP-3, doxorubicin makes H9c2 cells unresponsive to a key pro-survival factor with ensuing apoptosis. Further research work is needed to confirm these findings in human cardiomyocytes and to determine whether pharmacological manipulation of the myocardial IGF-1 system may be an effective strategy to prevent anthracycline cardiotoxicity.

## Supporting Information

S1 Fig Modulation of *Igf1r* and *Igfbp3* mRNA expression by doxorubicin. Expression of *Igf1r* and *Igfbp3* 24 hours after no treatment (Ctr) or incubation of H9c2 cardiomyocytes with 0.1, 0.5, or 1 μM doxorubicin (Dox). ***, P <0.001 vs. Ctr; c, P <0.001 vs. Dox 0.1; d, P <0.001 vs. Dox 0.5.
(TIF)

S2 Fig Doxorubicin elicits oxidative stress in cardiomyocytes. 2´,7´-dichlorofluorescein (DCF) production 24 hours after no treatment (Ctr) or incubation of H9c2 cells with 1 μM doxorubicin (Dox), preceded or not by N-acetylcysteine (NAC), dexrazoxane (DEX), or carvedilol (CARV). *, P <0.05 vs. Ctr. #, P <0.05 vs. Dox 1; ^, P <0.01 vs. Dox 1.
(TIF)
