# Supplementary figures and images for: Doxorubicin Impairs the Insulin-Like Growth Factor-1 System and Causes Insulin-Like Growth Factor-1 Resistance in Cardiomyocytes
Source: PLoS One. 2015 May 8;10(5):e0124643. doi: 10.1371/journal.pone.0124643 (PMC4425434; doi:10.1371/journal.pone.0124643)

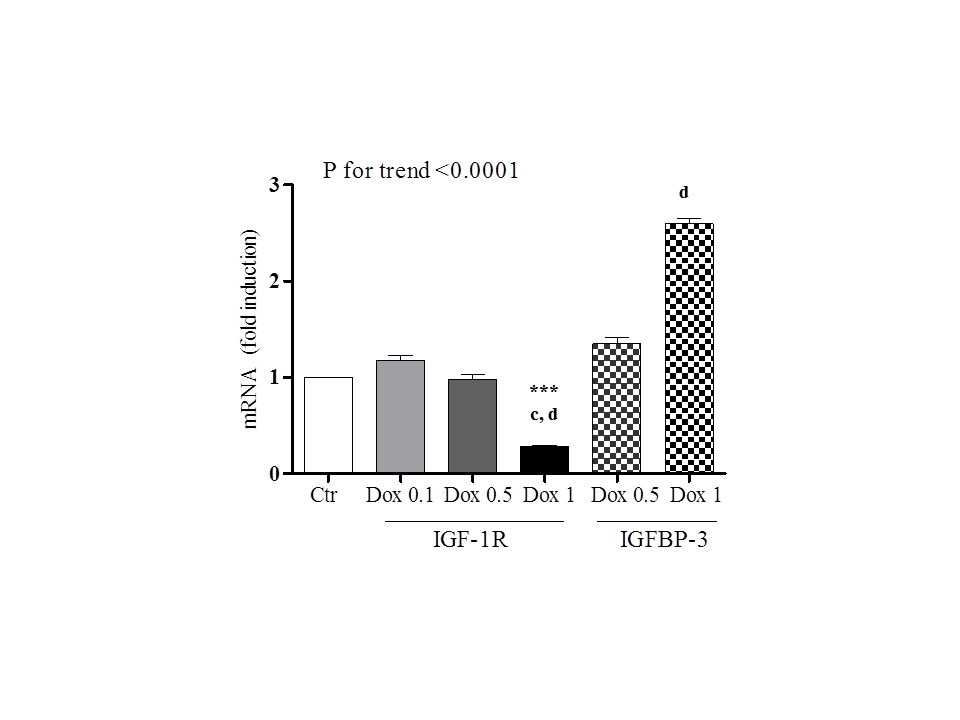

Supplement: S1 Fig — Expression of Igf1r and Igfbp3 24 hours after no treatment (Ctr) or incubation of H9c2 cardiomyocytes with 0.1, 0.5, or 1 μM doxorubicin (Dox). ***, P <0.001 vs. Ctr; c, P <0.001 vs. Dox 0.1; d, P <0.001 vs. Dox 0.5. (TIF) [file pone.0124643.s001.tif]

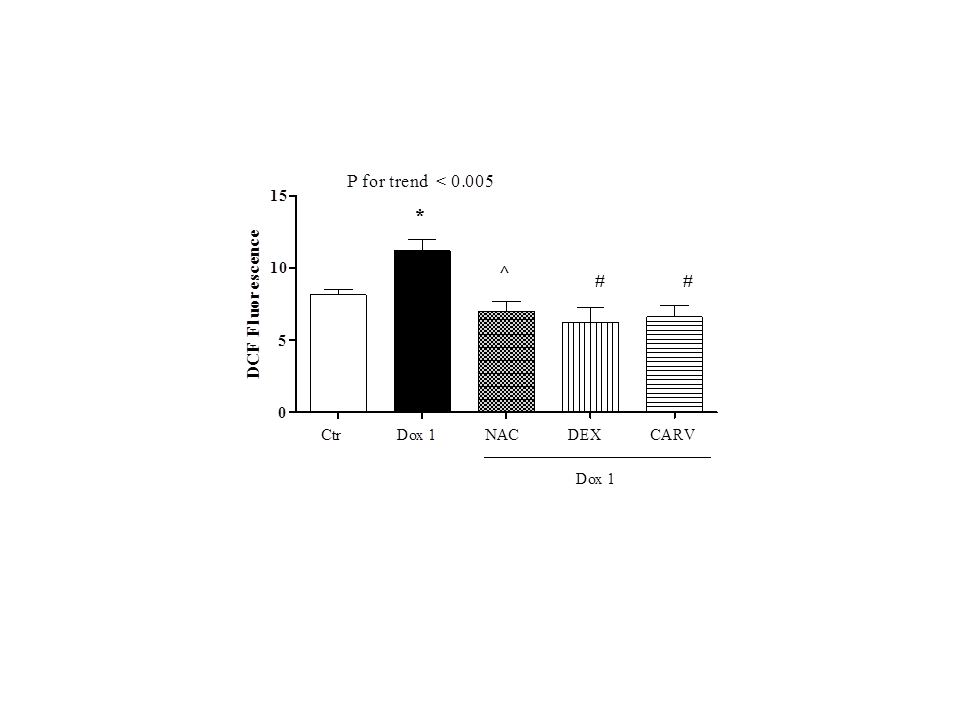

Supplement: S2 Fig — 2´,7´-dichlorofluorescein (DCF) production 24 hours after no treatment (Ctr) or incubation of H9c2 cells with 1 μM doxorubicin (Dox), preceded or not by N-acetylcysteine (NAC), dexrazoxane (DEX), or carvedilol (CARV). *, P <0.05 vs. Ctr. #, P <0.05 vs. Dox 1; ^, P <0.01 vs. Dox 1. (TIF) [file pone.0124643.s002.tif]
